# Effects of radiation based on whole-body irradiation in HTLV-1-infected mice

**DOI:** 10.1093/jrr/rrz050

**Published:** 2019-07-19

**Authors:** Masakazu Tanaka, Yusuke Kawazu, Toshinori Yoshida, Tomoko Konishi, Norihiro Takenouchi, Masanao Miwa

**Affiliations:** 1 Department of Microbiology, Kansai Medical University, Hirakata, Osaka, Japan; 2 Faculty of Bioscience, Nagahama Institute of Bioscience and Technology, 1266 Tamura, Nagahama, Shiga, Japan; 3 Division of Neuroimmunology, Joint Research Center for Human Retrovirus Infection, Kagoshima University, Kagoshima, Japan; 4 Laboratory of Veterinary Pathology, Tokyo University of Agriculture and Technology, 3-5-8 Saiwai-cho, Fuchu-shi, Tokyo, Japan

**Keywords:** human T-cell leukemia virus type-1, radiotherapy, animal model, whole-body irradiation

## Abstract

Adult T-cell leukemia is one of the life-threatening diseases that occur in individuals infected with human T-cell leukemia virus type 1 (HTLV-1). Clinical trials of hematopoietic stem cell transplantation therapy are being performed in addition to chemotherapy; however, neither is satisfactory. As a pretreatment for transplantation, anticancer drugs or whole-body irradiation is used to decrease the number of HTLV-1-infected cells, but there are numerous side effects. Therefore, in the present study, using a mouse model of HTLV-1 infection, the long-term survival and number of infected cells in the reservoir organ were investigated in order to determine the effect of γ-irradiation on HTLV-1-infected mice *in vivo*. There was no improvement in the survival period following γ-irradiation in the γ-irradiated group after HTLV-1 infection when compared with the HTLV-1-infected group. It was also found that the incidence of splenomegaly was ≥80% in the HTLV-1-infected and γ-irradiated group, which was significantly higher than that in the HTLV-1-infected mice. The tissue morphology in the spleen became non-uniform because of γ-rays. Importantly, the number of infected cells in the spleen was increased 4.1-fold in the HTLV-1-infected and γ-irradiated mice compared with that in the HTLV-1-infected mice. Careful consideration might be necessary when using whole-body irradiation in patients with HTLV-1 infection.

## INTRODUCTION

Human T-cell leukemia virus type 1 (HTLV-1) exists as a provirus in host cells following infection, and ~5% of HTLV-1 carriers become adult T cell leukemia (ATL) following 50–60 years of persistent infection [1, 2]. A higher proviral load in the peripheral blood is reported to be a risk factor for ATL [3]. For aggressive ATL, long-term survival is anticipated by VCAP-AMP-VECP therapy, combination therapy with human antibody CCR4 antibody (mogamurizumab), and allogeneic hematopoietic stem cell transplantation therapy.

The aim of radiation therapy is to completely destroy the tumor or to reduce tumor size, and it is used as a pretreatment prior to hematopoietic stem cell transplantation for ATL. However, the effect of irradiation on reduction of proviral loads in the reservoir organ is not well characterized. The present study used an HTLV-1-infected mouse model and examined the effects of whole-body γ-irradiation on HTLV-1-infected cells in the reservoir organs *in vivo* [4, 5].

## MATERIALS AND METHODS

### Cells and animals

MT-2 cells, an HTLV-1-infected human T-cell line, were cultured as described [6]. C57BL/6JJcl female mice at 5 weeks of age were purchased from Clea, Inc., Tokyo, Japan. The mice were inoculated intraperitoneally with 2.5 × 10^6^ MT-2 cells [4, 7]. The experiments were conducted in accordance with the Regulations on Animal Experiments of Kansai Medical University (Hirakata, Japan) and were approved by the University’s Animal Experiment Committee. γ-Ray irradiation (1.6 Gy) was performed four times from 1 week post-HTLV-1 infection at 1 week intervals (Gammacell 40 exactor・^137^Cs, Nordion International). The dose rate of the γ-irradiation was 0.952 Gy/min. Autopsy was performed when a reduction in body weight of ~30% or on day 260 after birth was observed, and tumors occurring in the thymus and spleen were excised.

### Histological examination

The spleens of mice were fixed in 10% neutral formalin and embedded in paraffin. The paraffin sections of 5 μm thickness were stained with hematoxylin and eosin and were examined microscopically.

### Deoxyribonucleic acid extraction

DNA from the spleen was extracted by sodium dodecyl sulfate-proteinase K digestion, followed by phenol extraction.

### Quantification of human T-cell leukemia virus type 1 proviral load

The polymerase chain reaction (PCR) conditions for quantification of the HTLV-1 proviral load were as described previously [8]. Briefly, the number of *tax* and mouse c-*myc* molecules were quantified using real-time PCR, and the HTLV-1 proviral load per 10^5^ mouse cells was calculated as follows: (number of tax molecules/number of mouse c-*myc* molecules/2) × 10^5^. The proviral load was defined as zero when there was no amplification of the *tax* product following 50 cycles of PCR under conditions where mouse c-*myc* was amplified correctly.

### Statistical analysis

Welch’s *t*-test was used to detect any difference between the mean scores in two groups, based on the equality test of two variances.

## RESULTS AND DISCUSSION

**Fig. 1.**
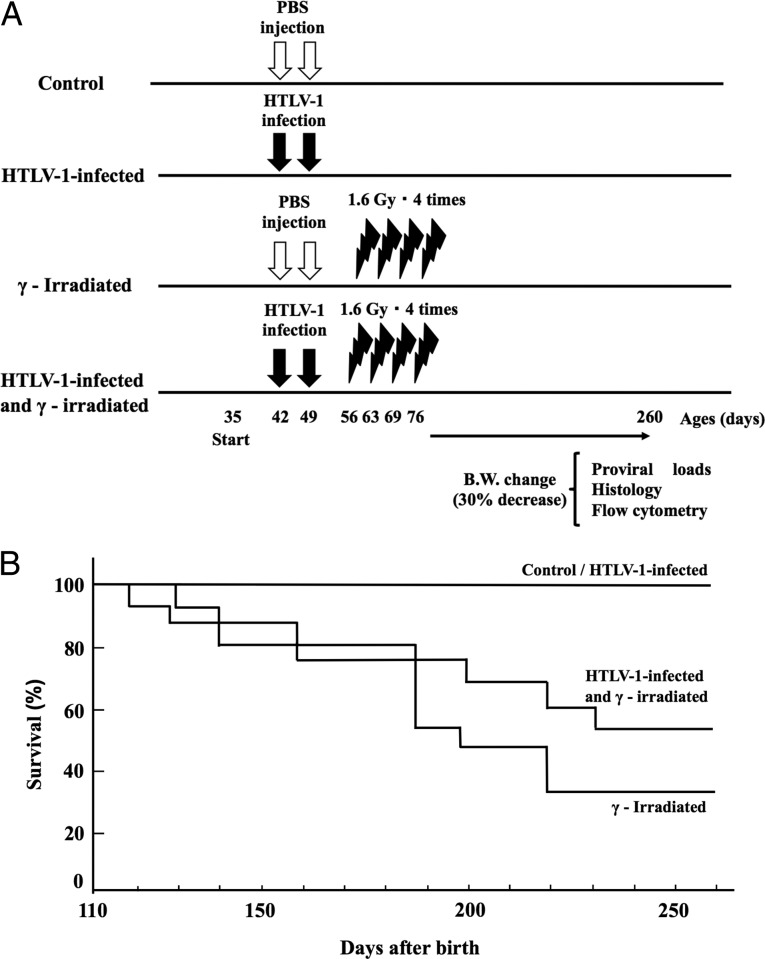
Schematic representation of the experimental protocol. (**A**) Mice were sacrificed when the body weight (B.W.) was reduced by 30%, or on day 260 after birth. Phosphate buffered saline (PBS) was a negative control. (**B**) Survival curves of mice in the untreated group (6 mice), HTLV-1-infected group (5 mice), irradiated group (15 mice), and HTLV-1-infected and γ-irradiated group (15 mice) are shown.

**Fig. 2.**
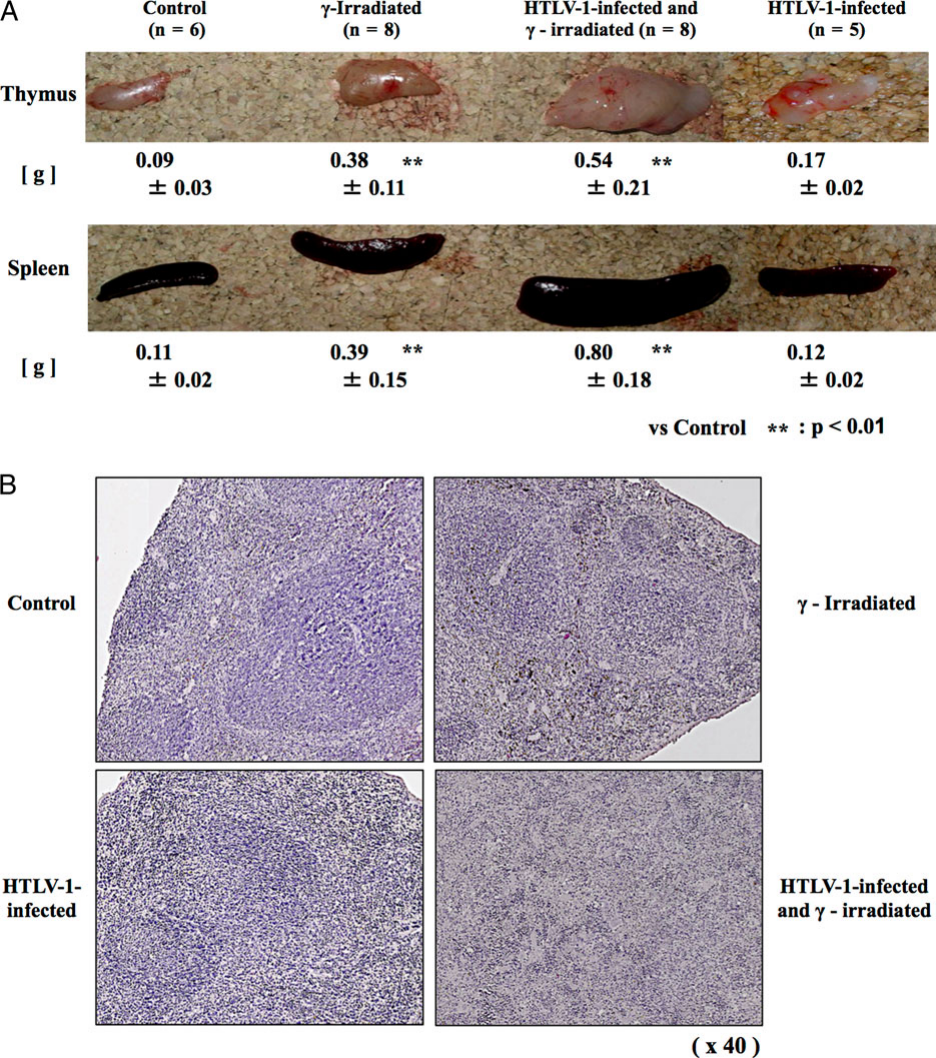
Histological characteristics of the thymus and the spleen. (**A**) Representative images of the thymus and spleen. Weights of each organ are shown as the average ± standard deviation. Significance was determined against the control group. (**B**) Histology of the spleen after hematoxylin and eosin staining (×40).

To investigate the effects of whole-body γ-irradiation on HTLV-1-infected mice, 42-day-old mice were infected. At 1 week post-infection, 1.6 Gy was irradiated four times (Fig. 1A). There was no significant difference in the survival curve between the HTLV-1-infected group and the HTLV-1-infected and γ-irradiated group (*P* = 0.099), and between the γ-irradiated group and the HTLV-1-infected and γ-irradiated group by a log-rank test (*P* = 0.276) (Fig. 1B). In the γ-irradiated group, thymoma was significantly observed in 13 out of 15 mice (*P* < 0.01) and splenomegaly was observed in three mice at 4 months in the 12 month observation period; in the HTLV-1-infected and γ-irradiated group, thymoma was observed in 2 out of 15 mice and splenomegaly was significantly observed in 13 out of 15 mice (*P* < 0.01) (Table 1). The weights of the thymus in the γ-irradiated group (0.38 g) and the HTLV-1-infected and γ-irradiated groups (0.54 g) were significantly higher than that of the control group (0.09 g), whereas the thymus weight in the HTLV-1-infected group remained unchanged (0.17 g). The weights of the spleen of the γ-irradiated group (0.39 g) and the HTLV-1-infected and γ-irradiated group (0.80 g) were significantly higher than that of the control group (0.11 g), whereas that of the HTLV-1-infected group remained unchanged (0.12 g) (Fig. 2A). Furthermore, the white pulp of the spleen was atrophic in the γ-irradiated group and HTLV-1-infected group and was lost in the HTLV-1-infected and γ-irradiated group (Fig. 2B). We previously showed that HTLV-1 can infect T cells in our mouse model of HTLV-1 infection [4], but splenomegaly was quite unpredicted and remarkable.

**Table 1.** Thymic lymphoma and splenomegaly

Group	No.	Thymic lymphoma	Splenomegaly
**Control**	**6**	**0**	**0**
**HTLV-1-infected**	**5**	**0**	**0**
**γ-Irradiated**	**15**	13**	**3**
**HTLV-1-infected and γ-irradiated**	**15**	**2**	13**

Tumor-generating organs (thymus/spleen) in each group. Significance is against control group. **P < 0.01.

It is known that thymoma occurs in mice by repeated γ-irradiation with 1 week intervals [9]. It is speculated that, during repeated regeneration of normal lymphocytes, abnormal lymphocytes that do not proliferate normally begin to proliferate, which may result in thymoma [10, 11]. Of note, splenomegaly occurred more frequently in the HTLV-1-infected and γ-irradiated group, and it may be that HTLV-1-infected T cells are reserved in the spleen and lymph nodes [4, 5]. Cancer cells generated in the thymus by γ-irradiation may accumulate in the spleen, eventually leading to splenomegaly.

Finally, the level of HTLV-1 provirus was compared between the HTLV-1-infected group and the HTLV-1-infected and γ-irradiated group. The assumption that HTLV-1-infected cells contain a single HTLV-1 provirus/cell was used. In the HTLV-1-infected group, 61.4 infected cells were present in 10^5^ cells in the spleen, whereas the number of infected cells was 37.9 in 10^5^ cells in the spleen of the HTLV-1-infected and γ-irradiated group, which was significantly lower (Table 2, upper). However, the weight of the spleen in the HTLV-1-infected and irradiated group was 6.7-fold higher than that in the HTLV-1-infected group (Fig. 2 and Table 2, lower). If the numbers of cells are proportional to the weight of the spleen, the number of HTLV-1-infected cells in the spleen was 4.1-fold higher in the HTLV-1-infected and γ-irradiated mice (Table 3). Thus, although γ-ray irradiation is considered to be useful for reducing HTLV-1-infected cells in humans, the reverse was true in the mouse model used in the present study. Further investigations are required to evaluate the effect of radiation on HTLV-1-infected cells *in vivo*.

**Table 2.** Proviral loads of HTLV-1 and the weight of the spleen

Group	HTLV-1-infected	HTLV-1-infected and γ-irradiated
Proviral loads/10^5^ cells^a^	61.4 ± 11.7	37.9 ± 5.9**
	(*n* = 8)	(*n* = 8)
Spleen weight (g)	0.12 ± 0.02	0.80 ± 0.18**
	(*n* = 5)	(*n* = 8)

^a^Proviral loads in 10^5^ cells and spleen weights. Weights of the spleen are shown as the average ± standard deviation. Significance is against HTLV-1-infected group. **P < 0.01.

**Table 3.** Relative ratio of spleen weight and HTLV-1-infected cells in the spleen

Group	HTLV-1-infected	:	HTLV-1-infected and γ-irradiated
Spleen weight	1	:	6.7
HTLV-1-infected cells*	1	:	4.1

*Ratio of HTLV-1-infected cells was multiplied by the ratio of the spleen weight (Table 2).

## ACKNOWLEDGEMENTS

We are indebted to Dr. Junichi Fujisawa (Kansai Medical University) for generous support of the work. We thank Enago (www.enago.jp) for the English language review.

## CONFLICT OF INTEREST

The authors declare that they have no conflicts of interest.

## FUNDING

This work was supported partly by KAKENHI Grant Number JP04J11896 from JSPS, and the Emerging/Re-emerging Infectious Diseases Project of Japan Grant Number JP17fk0108111j0101 from the Japan Agency for Medical Research and Development (AMED). The funders had no role in study design, data collection and analysis, decision to publish, or preparation of the manuscript.
